# Notes from the Rear Rank

**Published:** 1872-08

**Authors:** G. Newkirk

**Affiliations:** New Boston, Mo.


ARTICLE III. — *Notes from the Rear Rank.* By G. NEWKIRK, M.D., of New Boston, Mo.

CASE I. Caries of the tarsal bones of seventeen years' standing; amputation of leg.

May 5th, 1872. Mrs. Moss, aged 43, presented herself with a "sore foot," according to her custom, as she said, of consulting every "*new Doctor*" she could hear of.

Sanguine nervous temperament, with a good balance of the sarcous. Is much emaciated; general health poor; countenance presents the brown, dead hue peculiar to opium eaters; pulse slow and weak; blood poor; no organic lesion of heart or lungs.

Seventeen years ago received an injury of the left foot by falling from a horse. A "rising" ensued, which was opened with a free discharge of pus; this opening became permanent. Much medical and no surgical treatment was given by all sorts of "Fellows." The locality was known to the passer-by, from the number of elm trees girdled—for poultices!

The disease gradually extended itself from bone to bone, and the number of sinuses increased from one to fourteen, making a complete circle of discharging sores about the instep. For the past three years has borne no weight upon the limb; could secure rest only by the constant and excessive use of morphia; is in never-ending, never-ceasing pain.

The foot is considerably swollen, hard, and integument of a bluish dark color.

A probe passed into a sinus over the os cuboides passes readily through the foot to the opposite side, passed backward and forward reaches for some distance, encountering shells of bone here and there, some of which give way on pressure; the bases of the metatarsal bones are easily permeable. The examination is made with but little pain to the patient.

"*The Doctors*" have all lately pronounced the complaint "incurable," yet have refused to amputate or advise amputation for the reason given, that it is "*scrofulous*;" "the stump would not heal;" or, if it should, "something else would *set in*." Therefore the woman is placed in her present precarious condition by their ignorance and procrastination. She has to choose between the dangers of gangrene and sloughing with death, on the one hand, or the serious dangers of an operation in her present bad state of health, upon the other. An operation, however unfavorable cannot shorten life much, with the chances of lengthening it for years.

In consultation with Dr. Woods, of North Salem, an operation was advised, and all the risks presented. The patient wishes us to operate.

Tuesday, June 4th, assisted by Drs. Wood and Cox, I amputated the leg by the flap method, at the upper part of the middle third. Bone at point of operation sound; muscles soft, flabby and pale; arteries weak, pale and small; only the main three required ligation; very little loss of blood; very little oozing after dressing. About the third day thereafter, inflammation became active, but was quickly subdued by the application of continuous cold by water kept dripping upon the stump and toward the knee. Pain was controlled by morphia in grain doses; quinia, chlorate of potassa and iron were given, and a good nutritious diet provided. Mutton (in absence of beef) was administered in broth and substance as the stomach would bear, with stale bread, eggs, milk, wholesome canned fruits, etc., and an occasional weak brandy sling.

It was astonishing to witness the rapidity of constitutional reaction. With the removal of the grand cause of irritation came a vigorous and healthy general action. Digestion at once improved, with attendant appetite; the blood consequently became increased in volume and nutrient properties, and the heart became in a manner strong. At the end of four weeks the woman eats three hearty meals each day, and the stump is nearly healed. Carbolic acid and glycerine have constituted the local dressing.

An examination of the foot revealed almost total destruction of the os cuboides, scaphoid, astragalus and cuniform bones. The metatarsal were but shells of dead bones; the calcaneum retained its outward form, but was partly broken down through its whole extent; a small portion of the tibial joint surface had also experienced decay. The soft parts had undergone in part induration, and in part fatty degeneration, or substitution.

No term in medical nomenclature has been applied so indefinitely and unmeaningly as *scrofula*. It has been made to cover a greater multitude of physical sins than charity has of the spiritual. It is astonishing, almost, to witness the rapid improvement under a healthy regime—conducted with an eye single to the proper amount and quality of the blood—in those cases ordinarily denominated scrofulous. *Nutrition is the sheet anchor and our only hope;* all medication is but an *adjuvant* to that if it possess any value, and generally speaking the less of it the better.

CASE II. Mrs. F., aged 68, has been for years suffering from "gravel." Spasm of the bladder has occurred at short intervals, attended by great suffering. No exploration was ever made by the attending "Fellow," but constant medication employed, for "gravel," and "falling of the womb"!

Saw her, for the first time, June 22nd. A vesical calculus, 1 inch by 1¾ inches in diameter, has forced its way into the urethral canal and presents its smaller extremity at the orifice, which is tightly constricted upon the roughened surface of the stone. Being pushed back into the bladder, a pair of narrow forceps are made to grasp it near its middle portion; by gentle traction, and a rotatory motion (without which last nothing can be effected,) the stone is delivered in less than half a minute, and without injury to the parts. The woman immediately got up and went about. No inconvenience of any kind followed.

Comments on the previous "*treatment*" for "prolapsus uteri' are uncalled for.

